# EGFR-TKIs治疗晚期非小细胞肺癌获益后出现缓慢进展的治疗选择：附32例病例总结

**DOI:** 10.3779/j.issn.1009-3419.2013.10.05

**Published:** 2013-10-20

**Authors:** 琳 林, 彬 王, 学志 郝, 镨元 邢, 峻岭 李, 湘茹 张, 远凯 石

**Affiliations:** 100021 北京，中国医学科学院北京协和医学院肿瘤医院内科，抗肿瘤分子靶向药物临床研究北京市重点实验室 Department of Medical Oncology, Cancer Institute/Hospital, Chinese Academy of Medical Sciences & Peking Union Medical College; Beijing Key Laboratory of Clinical Study on Anticancer Molecular Targeted Drugs, Beijing 100021, China

**Keywords:** 肺肿瘤, EGFR-TKIs, 缓慢进展, 生存期, Lung neoplasms, EGFR-TKIs, Gradual progression, Overall survival

## Abstract

**背景与目的:**

表皮生长因子受体酪氨酸激酶抑制剂（epidermal growth factor receptor tyrosine kinase inhibitors, EGFR-TKIs）目前广泛应用于晚期非小细胞肺癌（non-small cell lung cancer, NSCLC），特别是存在表皮生长因子受体*EGFR*基因突变的肺腺癌患者。对于治疗后进展的患者，后续治疗未取得共识。本文总结EGFR-TKIs治疗后缓慢进展的晚期NSCLC患者接受不同后续治疗方法的近期疗效、毒性反应和总生存期，评价不同治疗方法的意义。

**方法:**

回顾性分析我院2003年9月-2011年12月期间32例接受EGFR-TKIs治疗后缓慢进展的晚期NSCLC患者，分别继续接受EGFR-TKIs治疗或化疗。

**结果:**

EGFR-TKIs维持治疗组患者的中位生存时间为36.0个月，在改行化疗的患者中，化疗有效率为43.75%，总的临床获益率（完全缓解+部分缓解+稳定）为87.5%。中位生存时间为15.5个月。主要的毒性反应为恶心呕吐等消化道反应和血液学毒性。

**结论:**

在EGFR-TKIs治疗后出现肿瘤缓慢进展的患者中，维持原EGFR-TKIs治疗是可行的选择。

## Abstract (en)

**Background and objective:**

The epidermal growth factor receptor tyrosine kinase inhibitors (EGFR-TKIs) have been widely used in the treatment of the advanced non-small cell lung cancer (NSCLC), especially in the adenocarcinoma patients with activating *EGFR* mutations. But there is no published overview of the following treatment. This report through observing the efficacy, toxicity and overall survival of different treatments to the advanced NSCLC patients who had gradual progression after EGFR-TKIs, evaluates the influence of the continued treatment and switching chemotherapy.

**Methods:**

Retrospective review is conducted on 32 cases of advanced NSCLC patients who experienced treatment failure of EGFR-TKIs. One group accepted the continued treatment and the other group accepted the switching chemotherapy.

**Results:**

The median overall survival of the continued treatment group is 36.0 months. The respose rate of the switching chemotherapy group is 43.75%, and clinical benefit rate (complete and partial response and stable disease) is 87.5%. The median overall survival is 15.5 months. The main toxicities are nausea, vomiting and hematological toxicities.

**Conclusion:**

For the advanced NSCLC patients who had gradual progression after EGFR-TKIs, the continued treatment is one of the acceptable choices.

肺癌是全球范围内肿瘤相关死亡的第一位死因，其中非小细胞肺癌（non-small cell lung cancer, NSCLC）约占85%^[1, 2]^。初诊时，大约70%的NSCLC患者属于局部晚期或已发生远地转移，含铂方案的联合化疗可以延长生存，改善症状，中位生存时间在8个月-10个月^[3]^。表皮生长因子受体酪氨酸激酶抑制剂（epidermal growth factor receptor tyrosine kinase inhibitors, EGFR-TKIs）上市之后，迅速成为晚期NSCLC特别是腺癌不可或缺的治疗方法，明显改善了部分患者的预后和生存，在存在*EGFR*基因敏感突变的晚期肺腺癌患者中更是取得了优于化疗的治疗疗效^[4, 5]^。

然而，对于初次接受EGFR-TKIs治疗有效的患者，在治疗过程中不可避免会出现因耐药而导致的治疗失败。有学者将EGFR-TKIs治疗失败分为三种类型，即肿瘤爆发式进展、肿瘤缓慢进展及肿瘤局部进展^[6]^。目前，对于EGFR-TKIs耐药后的治疗并无指南共识，治疗多以化疗为主，也有患者二次接受EGFR-TKIs药物治疗的报道^[7]^。对于合并孤立脑转移或骨转移的患者，亦有继续EGFR-TKIs治疗同时联合局部放疗的报道^[8]^。本文通过回顾性分析我院内科接受EGFR-TKIs治疗后发生肿瘤缓慢进展的晚期NSCLC患者的临床资料，结合文献，观察治疗进展后的患者继续服用EGFR-TIKs或改行化疗的不同治疗疗效，为今后的治疗提供更多的信息和选择。

## 1 材料与方法

### 1.1 病例收集

回顾性分析中国医学科学院肿瘤医院自2003年9月-2011年12月就诊的晚期NSCLC患者的临床资料，选择EGFR-TKIs治疗有效且治疗持续时间超过6个月，肿瘤发生进展，但是与EGFR-TKIs初始治疗时相比，肿瘤负荷进展不明显，且没有明显临床症状的患者，定义为肿瘤缓慢进展。选取其中继续维持EGFR-TKIs原药治疗，后续无化疗或改行化疗，后续不再接受EGFR-TKIs药物再次治疗的患者资料，共计32例。

### 1.2 患者特征

维持原EGFR-TKIs药物治疗组（continued treatment）16例，中位年龄58岁（38岁-72岁）；男性9例，女性7例；全部为Ⅳ期患者；腺癌15例，鳞癌1例；行为状态ECOG评分1分-2分，其中ECOG 1分15例，ECOG 2分1例；服用易瑞沙10例，特罗凯6例；存在外显子19突变3例，外显子21突变3例，10例基因情况不明；一线及二线EGFR-TKIs治疗者13例，三线及以上者3例；中位治疗持续有效时间14.0个月（6.1个月-29.0个月）。换行化疗组（switching chemotherapy）16例，中位年龄59岁（43岁-76岁）；男性7例，女性9例；全部为Ⅳ期患者；全部为腺癌；行为状态ECOG评分1分-2分，其中ECOG 1分13例，ECOG 2分3例；服用易瑞沙10例，特罗凯6例；存在外显子19突变4例，外显子21突变1例，11例基因情况不明；一线及二线EGFR-TKIs治疗者12例，三线及以上者4例；中位治疗持续有效时间16.0个月（8.2个月-26.0个月）。一般临床资料见表 1。

**1:** 患者特征 Basic characteristics of the patients

Total	*n*	Proportion (%)
Gender		
Male	16	50.000
Female	16	50.000
Age（yr）		
Median	59	
Range	38-76	
Histological type		
Squamous cell carcinoma	1	3.125
Adenocarcinoma	31	96.875
*EGFR* mutation		
Exon 19	7	21.875
Exon 21	4	12.500
Not available	21	65.625
Duration of TKIs (month)		
Median	15	
Range	6.1-29.0	
Treatment options after EGFR-TKIs failure		
Continued EGFR-TKIs	16	50.000
Switching chemotherapy	16	50.000
EGFR-TKI: epidermal growth factor receptor tyrosine kinase inhibitor.		

### 1.3 随诊和统计方法

随诊截止至2013年5月20日。统计分析采用SPSS 17.0软件，使用*Kaplan-Merier*法计算生存时间和生存率，绘制生存曲线。

## 2 结果

### 2.1 治疗方法

所有维持EGFR-TKIs治疗患者均继续原药治疗，吉非替尼用法250 mg/d，特罗凯150 mg/d，仍保持每8周复查1次，观察病情变化。换行化疗患者的治疗方案中包含培美曲塞、吉西他滨、多西他赛、卡铂、奈达铂、贝伐珠单抗、西妥昔单抗等药物。患者每2周期化疗后复查1次，评价疗效。如果达到完全缓解（complete response, CR）、部分缓解（partial response, PR）或稳定（stable disease, SD），根据患者的意愿和体力状况，可以继续维持化疗，否则结束治疗；疾病再次进展后换方案继续化疗。

### 2.2 评价指标

#### 2.2.1 化疗组近期疗效及无进展生存

疗效评价采用RECIST实体肿瘤评价标准，包括CR、PR、SD和PD。无进展生存（progression free survival, PFS）定义为化疗开始至肿瘤进展的时间。

#### 2.2.2 观察两组患者的总生存（overall survival, OS）

生存期定义为EGFR-TKIs治疗进展至患者死亡的时间。

### 2.3 化疗组近期疗效及无进展生存

全部患者未出现CR病例。PR 7例（43.75%），SD 7例（43.75%），PD 2例（12.5%），总的临床获益率（CR+PR+SD）为87.5%。中位无进展生存期4.7个月（1.4个月-19.3个月）。

### 2.4 总生存

患者末次随诊为2013年5月20日，至末次随诊，无失随。目前维持治疗组12例患者存活，中位生存时间36.0个月。化疗组2例患者尚未出现疾病进展，8例患者存活，中位生存时间15.5个月（图 1）。

**1:**
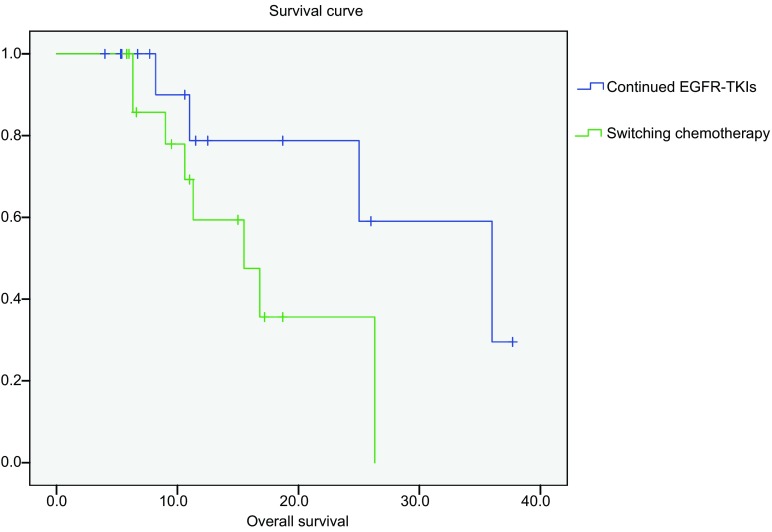
不同治疗组总生存 Overall survival of two groups

### 2.5 毒性反应

继续EGFR-TKIs治疗的患者耐受性良好，不良反应较轻，包括1度-2度皮疹6例，3例患者出现间断性腹泻，对症处理后可完全缓解。2例患者出现一过性肝功能损伤，经治疗后好转，未出现因不良反应停药的患者。改行化疗的患者出现的毒性反应包括骨髓抑制，3度-4度骨髓抑制发生率为40%-50%，1度-2度胃肠道反应80%，偶见肝功能损伤、乏力、脱发等，均可耐受，无化疗相关死亡发生。经对症处理后完全缓解。

## 3 讨论

EGFR-TKIs治疗失败的晚期NSCLC的治疗是目前研究的焦点和热点，但是仍未取得共识。而且，因为EGFR-TKIs治疗失败的模式缺乏明确的定义，所以对于不同类型的进展方式的后续治疗也存在较大的差异。因此，笔者认为，将EGFR-TKIs治疗失败患者按不同类型分别对待，进行个体化治疗十分必要。

本研究回顾性分析了32例EGFR-TKIs治疗后缓慢进展的晚期NSCLC患者的临床资料，分别剔除化疗或二次EGFR-TKIs治疗的影响，仅关注于EGFR-TKIs治疗或化疗本身的作用。可以看到，在基线水平，两组患者在各个方面的情况都是基本均衡的，在疾病进展以后，通过不同的治疗，结果发现，尽管EGFR-TKIs维持治疗不能阻止肿瘤的进一步进展，但是肿瘤进展是缓慢而可控的，其中位生存时间达到了36个月。Asami^[9]^的研究提示，对于存在基因敏感突变的易瑞沙治疗失败患者，继续采用易瑞沙治疗，中位生存时间为14.3个月，可以获得较长时间的临床生存获益。与该研究相比，本研究的结果似乎更好，这可能是因为我们只选择了疾病缓慢进展的晚期NSCLC患者加以研究；而对于疾病爆发式进展的患者，EGFR-TKIs药物维持治疗是否适合还有待于进一步的研究证实。另外，对于那些观察病灶控制良好，但因为出现骨转移或脑转移导致进展的患者，其治疗模式也期待进一步的研究给出答案。在化疗组，临床有效率达到43.75%，中位PFS为4.7个月，与临床上广泛应用的有效的一线NSCLC治疗方案疗效相似^[10, 11]^，也提示我们在EGFR-TKIs治疗失败后，化疗，特别是联合其他靶向药物的化疗，也可以发挥很好的治疗作用。有研究^[7]^关注于初始EGFR-TKIs治疗耐药后再次使用EGFR-TKIs的疗效，结果发现，再次使用EGFR-TKIs治疗的有效率为21.7%，疾病控制率为65.2%。这样的研究提醒临床医生，在化疗后，再次选择EGFR-TKIs治疗，对既往治疗有效的患者，可能带来进一步的生存获益。

在这项回顾性研究中可以看到，与治疗失败后仅接受化疗相比，即使单纯继续EGFR-TKIs治疗，患者也能获得更久的生存获益，因此，对于那些既往EGFR-TKIs治疗有效时间长，肿瘤进展不明显且没有明显临床症状的患者，适当延长EGFR-TKIs的治疗时间，或者在化疗基础上合并EGFR-TKIs治疗，或者化疗后再次选择EGFR-TKIs治疗都将是可行的选择。

本研究为回顾性研究，病例数较少，得到的结论可能不够全面。但是我们可以从中看到EGFR-TKIs药物治疗带给患者的生存获益，为医生的临床工作提供一定的治疗信息。目前已有的研究均为小样本的回顾性分析，我们期待更大样本量的前瞻性研究结果进一步证实。

## References

[1] Herbst RS, Heymach JV, Lippman SM (2008). Lung cancer. N Engl J Med.

[2] Jemal A, Siegel R, Xu J (2010). Cancer statistics, 2010. CA Cancer J Clin.

[3] Georgoulias V, Ardavanis A, Tsiafaki X (2005). Vinorelbine plus cisplatin versus docetaxel plus gemcitabine in advanced non-small cell lung cancer: a phase Ⅲ randomized trial. J Clin Oncol.

[4] Mok TS, Wu YL, Thongprasert S (2009). Gefitinib or carboplatin-paclitaxel in pulmonary adenocarcinoma. N Engl J Med.

[5] Mitsudomi T, Morita S, Yatabe Y (2010). Gefitinib versus cisplatin plus docetaxel in patients with non-small-cell lung cancer harboring mutations of the epidermal growth factor receptor (WJTOG3405): an open label, randomized phase 3 trial. Lancet Oncol.

[6] Wu YL, Yang JJ, Chen HJ (2013). Clinical modes of EGFR tyrosine kinase inhibitor failure and subsequent management in advanced non-small cell lung cancer. Lung Cancer.

[7] Oh IJ, Ban HJ, Kim KS (2012). Retreatment of gefitinib in patients with non-small-cell lung cancer who previously controlled to gefitinib: A single-arm, open-label, phase Ⅱ study. Lung Cancer.

[8] Inomata M, Shukuya T, Takahashi T (2011). Continuous administration of EGFR-TKIs following radiotherapy after disease progression in bone lesions for non-small cell lng cancer. Antic Res.

[9] Asami K, Okuma T, Hirashima T (2013). Continued treatment with gefitinib beyond progressive disease benefits patients with activating *EGFR* mutations. Lung Cancer.

[10] Giorgio VS, Purvish P, Joachim von Pawel (2008). Phase Ⅲ study comparing cisplatin plus gemcitabine with cisplatin plus pemetrexed in chemotherapy-naïve patients with advanced-stage non-small-cell lung cancer. J Clin Oncol.

[11] Fisher J, D'Orazio A (2000). Phase Ⅱ and Ⅲ trial: comparison of four chemotherapy regimens in advanced non-small cell lung cancer (ECOG 1594). Clin Lung Cancer.

